# Management of Alopecia Areata With Topical JAK Inhibitor Therapy: An Evidence-Based Review

**DOI:** 10.1177/12034754221130243

**Published:** 2022-10-02

**Authors:** Abrahim Abduelmula, Asfandyar Mufti, Jenna Mistry, Muskaan Sachdeva, Jennifer Beecker, Vimal H. Prajapati, Jensen Yeung

**Affiliations:** 1 6221 Faculty of Medicine, University of Western Ontario, London, Ontario, Canada; 2 7938 Division of Dermatology, Department of Medicine, University of Toronto, Ontario, Canada; 3 3710 Faculty of Health Sciences, McMaster University, Hamilton, Ontario, Canada; 4 Probity Medical Research, Waterloo, Ontario, Canada; 5 6363 Division of Dermatology, University of Ottawa, Ottawa, Ontario, Canada; 6 70401 Division of Dermatology, Department of Medicine, University of Calgary, Calgary, Alberta, Canada; 7 Dermatology Research Institute, Calgary, Alberta, Canada; 8 Skin Health & Wellness Centre, Calgary, Alberta, Canada; 9 Probity Medical Research, Calgary, Alberta, Canada; 10 Section of Community Pediatrics, Department of Pediatrics, University of Calgary, Alberta, Canada; 11 Section of Pediatric Rheumatology, Department of Pediatrics, University of Calgary, Alberta, Canada; 12 Women’s College Research Institute, Women’s College Hospital, Toronto, Ontario, Canada; 13 Sunnybrook Health Sciences Centre, Toronto, Ontario, Canada

**Keywords:** Alopecia, JAK inhibitor, treatment, therapeutics, topical, systematic review, evidence-based

Alopecia areata (AA) is a genetic, immune-mediated disease that targets hair follicles, resulting in patches of hair loss, loss of all scalp hair (alopecia totalis), and/or body hair (alopecia universalis). AA affects up to 2% of the general population.^1^ The use of topical Janus kinase inhibitors (JAKi) for AA remains experimental without FDA approval or consensus on their treatment efficacy. This systematic review examines the literature and summarizes the effectiveness and safety outcomes of topical JAKi therapies in the treatment of AA.

Following the PRISMA guidelines, MEDLINE and Embase Ovid searches were conducted, using variations of keywords “Alopecia” and “JAK” (Supplemental Table 2). Quality of evidence was assessed using Oxford Centre for Evidence-Based Medicine 2011 Levels of Evidence. After independent screening of 240 articles by two reviewers, 12 studies (publication date: 2017-2022) involving 136 patients were included (Supplemental Figure 1; Supplemental Table 3). The mean age was 37.6 years (range: 6-68 years), with 54 males (39.7%) and 78 females (57.4%); gender was not stated for 2.9% of patients. The reported subtypes of alopecia included alopecia areata (111/136, 81.6%), alopecia universalis (18/136, 13.2%), and alopecia totalis (7/136, 5.1%).

A total of 136 instances of topical JAKi use with outcomes were documented with treatments categorized as monotherapy (135/136, 99.3%) or combination therapy (1/136, 0.7%). Tofacitinib (59/135,43.7%) was the most common monotherapy, followed by ruxolitinib (56/135,41.5%), and delgocitinib (20/135,14.8%) (Supplemental Table 1). Treatment duration was described in 107 instances (mean: 142.6 days; range: 28-330 days). Severity of Alopecia Tool (SALT) scores were measured in 39% of cases (53/136), with a 24.9% reduction from baseline SALT and 23.5% (32/136) of cases achieving ≥50% (SALT50) reductions from baseline SALT. AA recurrence data with follow-up was available in 11% (15/136) of cases, with AA recurrence in 13.3% (2/15) of cases with a mean recurrence time of 2.3 months (range: 2.1-3). Concurrent treatment was not noted in any cases (Supplemental Table 3). Treatment-related adverse events occurred in 5 cases (3.7%), with no treatment discontinuation, specifically scalp skin irritation (4/5) and folliculitis (1/5) (Supplemental Table 1).

While the use of oral JAKi has shown potential as a successful treatment for AA, oral JAKi have been associated with side effects, such as infections, viral reactivation and rarely thromboembolic events.^2^ The appeal of topical JAKi is potentially a reduced side effect profile through a different mode of entry.^3^ Tofacitinib resulted in SALT50 in 32.2% of cases. This efficacy may be explained through tofacitinib’s method of action, which involves selectivity for JAK 1/3. This JAK inhibition results in the blocking of cytokine release (IL-2,4,7,15,21 and IFNγ) disrupting autoreactive *T* cell activation responsible for the pathogenesis of AA.^4^ Improved efficacy maybe be seen with longer treatment time, concurrent treatments for AA, and advancements in topical formulations increasing dermal penetration.^5^

Study limitations included a lack of follow-up and recurrence data. Due to limited sample size and heterogeneous outcome data, a meta-analysis could not be performed. Despite the low efficacy of topical JAKi for AA highlighted in the study, more rigorous and long-term studies are warranted to further determine the effectiveness and safety outcomes.

## Supplemental Material

Figure S1 - Supplemental material for Management of Alopecia Areata With Topical JAK Inhibitor Therapy: An Evidence-Based Review Click here for additional data file. Supplemental material, Figure S1, for Management of Alopecia Areata With Topical JAK Inhibitor Therapy: An Evidence-Based Review by Abrahim Abduelmula, Asfandyar Mufti, Jenna Mistry, Muskaan Sachdeva, Jennifer Beecker, Vimal H. Prajapati and Jensen Yeung in Journal of Cutaneous Medicine and Surgery
