# Supplementary material for: Management of Alopecia Areata With Topical JAK Inhibitor Therapy: An Evidence-Based Review
Source: J Cutan Med Surg. 2022 Oct 2;27(1):73–5. doi: 10.1177/12034754221130243 (PMC9902965; doi:10.1177/12034754221130243)
Supplement: Figure S1 - Supplemental material for Management of Alopecia Areata With Topical JAK Inhibitor Therapy: An Evidence-Based Review [file sj-pdf-1-cms-10.1177_12034754221130243.pdf]

Management of Alopecia with Topical JAK Inhibitor Therapy: An Evidence-Based Review

<u>**Supplemental Material**</u>

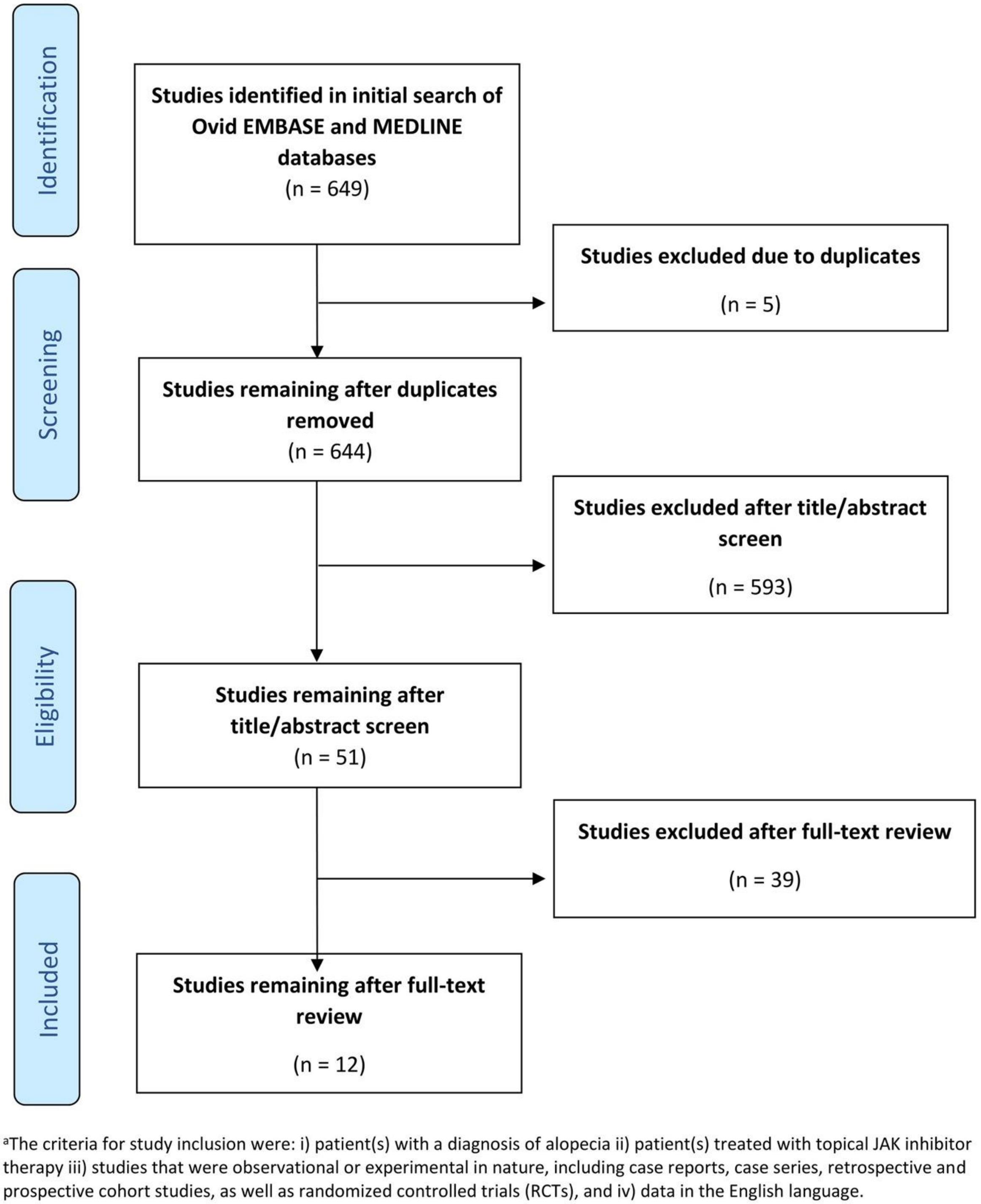

Figure S1. Flow diagram of literature screening using the Preferred Reporting Items for Systematic Reviews and Meta-Analyses (PRISMA) guidelines. Figure adapted from http://prisma-statement.org.

Table S1. Outcomes of topical JAK inhibitor therapy use for Alopecia. Abbreviations; BSA, Body Surface Area; CR, complete resolution; N, no, NR: none reported; PR, partial resolution; NOR, No resolution; SALT Severity of Alopecia Tool; Y, yes. Additional details on topical JAK inhibitor therapy dosing, vehicle , concomitant therapy use, baseline SALT score, and time to achieve SALT changes are listed in Supplemental File-2.

Treatment group (%, n/N)	Topical JAK inhibitor therapy (%, n/N)	Study design (n/N)	Treatment outcome (%, n/N)	Alopecia sub-type (%, n/N)	Mean change in SALT measures from baseline in % (n/N)	Treatment duration, days(n/N)	Recurrence (n/N)	Adverse events (n/N)	Mean follow-up period, months (n/N)
**Monotherapy (99.3%, 135/136)**	Tofacitinib (43.7%, 59/135)	Cohort study (2/8)<br>Case series (2/8)<br>Case report (2/8)<br>Randomized controlled trial (1/8)<br>Retrospective study (1/8)	CR (35.6%, 21/59)	Alopecia areata (81%, 17/21)<br>Alopecia universalis (14.3, 3/21)<br>Alopecia totalis (4.8%, 1/21)	-91.6% (4/21)	125 (18/21)	N (4/21)	NR	4 (1/21)
			PR (54.2%, 32/59)	Alopecia areata (71.9%, 23/32)<br>Alopecia universalis (29.1%, 7/32)<br>Alopecia totalis (6.3%, 2/32)	-51.9% (15/32)	121.8 (14/32)	N (6/32)<br>Y (1/32)	Scalp skin irritation (4/10); folliculitis (1/10)	2.1 (12/32)
			NOR (10.2%, 6/59)	Alopecia totalis (50%, 3/6)<br>Alopecia universalis (50%, 3/6)	76.1% (5/6)	NR	NR	NR	NR
	Ruxolitinib (41.5%, 56/135)	Randomized controlled trial (2/5)<br>Cohort study (1/5)<br>Case report (1/5)<br>Case series (1/5)	CR (14.3%, 8/56)	Alopecia areata (100%, 8/8)	-50% (8/8)	180 (8/8)	NR	NR	NR
			PR (12.5%, 7/56)	Alopecia universalis (57.1%, 4/8)<br>Alopecia areata (42.9%, 3/8)	NR	114.3 (6/7)	Y (1/7)	NR	3 (2/7)
			NOR (73.2%, 41/56)	Alopecia areata (97.6%, 40/41)<br>Alopecia totalis (2.4%, 1/41)	-6.9% (1/41)	180 (41/41)	NR	NR	NR
	Delgocitinib (14.8%, 20/135)	Randomized controlled trial (1/1)	NOR (100%, 20/20)	Alopecia areata (100%, 20/20)	-7.5% (20/20)	90 (20/20)	NR	NR	NR
**Combination therapy (0.7%, 1/136)**	Ruxolitinib and tofacitinib (100%, 1/1)	Cohort study (1/1)	NOR (100%, 1/1)	Alopecia universalis (100%, 1/1)	NR	NR	NR	NR	NR

Table S2. Search strategy used for literature screening.

Database(s):
Ovid MEDLINE: Epub Ahead of Print, In-Process & Other Non-Indexed Citations, Ovid MEDLINE® Daily and Ovid MEDLINE® 1946-Present, Embase Classic+Embase 1947 to 2022 April 20

Search strategy:

#	Searches	Results
1	alopecia.mp. [mp=ti, ab, hw, tn, ot, dm, mf, dv, kf, fx, dq, nm, ox, px, rx, an, ui, sy]	87765
2	ruxolitinib.mp. [mp=ti, ab, hw, tn, ot, dm, mf, dv, kf, fx, dq, nm, ox, px, rx, an, ui, sy]	9895
3	tofacitinib.mp. [mp=ti, ab, hw, tn, ot, dm, mf, dv, kf, fx, dq, nm, ox, px, rx, an, ui, sy]	9354
4	delgocitinib.mp. [mp=ti, ab, hw, tn, ot, dm, mf, dv, kf, fx, dq, nm, ox, px, rx, an, ui, sy]	142
5	JAK*.mp. [mp=ti, ab, hw, tn, ot, dm, mf, dv, kf, fx, dq, nm, ox, px, rx, an, ui, sy]	122707
6	2 or 3 or 4 or 5	132413
7	1 and 6	925
8	limit 7 to english language	892
9	remove duplicates from 8	649

Table S3. Cases of alopecia areata treated with topical JAK inhibitors.

Abbreviations: AE, adverse event; BID, twice daily; CAS, case series; COH, cohort study; CR, complete resolution; CRS, case report; CS, corticosteroids; F, female; HCQ, hydroxychloroquine; M, male; N, no; NOR, no response; NR, none reported; QD, once daily; PR, partial response; RCT, randomized controlled trial; RS, retrospective study; SALT, Severity of Alopecia Tool; TOP, topical; Y, yes.

Study	Study type (level of evidence)	Sample size of study extracted	Mean age (range), years	Sex (n)	Comorbidities (n)	Mean duration, years	Type of alopecia, years	Failed prior topical therapies	Concurrent topical therapies [route, dose, and frequency] (n)	Concurrent non-JAK systemic therapies [route, dose, and frequency] (n)	Topical JAK inhibitors [route, dose, and frequency] (n)	SALT pre- /post-treatment	Change in SALT score, %	Outcomes for topical JAK inhibitors: CR, PR, or NOR] (n)	Resolution period for topical JAK inhibitors, months	Recurrence despite topical JAK inhibitors	AEs for topical JAK inhibitors (n)	Follow-up period, months
Bayart 2017[1]	COH (2b)	1	13	M	Hashimoto thyroiditis	11	Alopecia universalis	CS	NR	NR	Tofacitinib 2% [ointment, BID] (1)	NR/ NR	NR	PR (1)	NR	NR	NR	NR
Bayart 2017[1]	COH (2b)	1	4	M	NR	1	Alopecia universalis	CS	NR	NR	Ruxolitinib 1% [ointment, BID] and tofacitinib 2% [ointment, BID] (1)	NR/ NR	NR	NOR (1)	NR	NR	NR	NR
Bayart 2017[1]	COH (2b)	1	17	F	NR	14	Alopecia universalis	CS	NR	NR	Ruxolitinib 1% [ointment, BID] (1)	NR/ NR	NR	PR (1)	NR	NR	NR	NR
Bayart 2017[1]	COH (2b)	1	15	F	Mild anemia	1	Alopecia areata	CS	NR	NR	Tofacitinib 2% [ointment, BID] (1)	NR/ NR	NR	PR (1)	NR	NR	NR	NR
Bayart 2017[1]	COH (2b)	1	3	F	Hemolytic disease of the newborn	1.5	Alopecia totalis	NR	NR	NR	Tofacitinib 2% [ointment, BID] (1)	NR/ NR	NR	PR (1)	NR	NR	NR	NR
Bayart 2017[1]	COH (2b)	1	5	F	NR	3.5	Alopecia totalis	CS; minoxidil	NR	NR	Tofacitinib 2% [ointment, BID] (1)	NR/ NR	NR	NOR (1)	NR	NR	NR	NR
Bokhari 2020[2]	RCT (1b)	4	NR	NR	NR	NR	Alopecia universalis	NR	NR	NR	Ruxolitinib 1% [ointment, BID] and tofacitinib 2% [ointment, BID] (4)	NR/ NR	NR	PR (4)	28	Y (2)	NR	3
Cheng 2018[3]	CAS (4)	1	28	M	NR	3.5	Alopecia universalis	NR	NR	NR	Tofacitinib 2% [ointment, BID] (1)	NR/ NR	NR	PR (1)	330	NR	NR	NR
Cheng 2018[3]	CAS (4)	1	28	M	NR	7	Alopecia universalis	CS; minoxidil	NR	NR	Tofacitinib 2% [ointment, BID] (1)	75/ 6.7	-91.1%	PR (1)	270	NR	NR	NR
Cheng 2018[3]	CAS (4)	1	56	M	NR	2	Alopecia universalis	Contact immunotherapy, ILK, clobetasol, oral tofacitinib 5mg BID	NR	NR	Tofacitinib 2% [ointment, BID] (1)	100/ 100	0	NOR (1)	90	NR	NR	NR
Cheng 2018[3]	CAS (4)	1	58	F	NR	10	Alopecia universalis	minoxidil	NR	NR	Tofacitinib 2% [ointment, BID] (1)	20/ 99	395%	NOR (1)	150	NR	NR	NR
Craiglow 2016[4]	CRS (5)	1	19	F	NR	2	Alopecia universalis	prednisone, intralesional triamcinolone,	NR	NR	Ruxolitinib 0.6% [ointment, BID] (1)	NR/ NR	NR	PR (1)	90	NR	NR	NR
Craiglow 2018[5]	CRS (5)	1	28	F	NR	0.9	Alopecia areata	CS; tacrolimus 0.1%	NR	NR	Tofacitinib 2% [ointment, BID] (1)	NR/ NR	NR	PR (1)	120	N (1)	NR	NR
Deeb 2017[6]	CAS (4)	1	66	F	NR	4	Alopecia totalis	NR	NR	NR	Ruxolitinib 0.6% [ointment, BID] (1)	29/ 27	-6.9%	NOR (1)	180	N (1)	NR	NR

Ferreira 2020[7]	CRS (5)	1	23	F	NR	12	Alopecia areata	NR	NR	NR	Tofacitinib 2% [ointment, QD] (1)	NR/ NR	NR	CR (1)	60	N (1)	NR	4
Kerkemeyer 2021[8]	RS (2b)	26	30 (6-54)	M (17); F (9)	NR	1.5	Alopecia areata	NR	NR	NR	Tofacitinib 2% [gel, QD] (26)	NR/ NR	NR	CR (16); PR (10)	120	NR	NR	NR
Liu 2018[9]	COH (2b)	10	36.9 (19-58)	M (6); F (4)	NR	9.4	Alopecia areata	NR	NR	NR	Tofacitinib 2% [ointment, BID] (10)	77.7/ 27.6	-64.5%	PR (10)	NR	NR	Scalp skin irritation (4), folliculitis (1)	2
Mikhaylov 2022[10]	RCT (1b)	20	36.4 (18-64)	M (6); F (14)	NR	5	Alopecia areata	NR	NR	NR	Delgocitinib 0.5% [ointment, BID] (20)	67/ 61.98	-7.5%	NOR (20)	90	NR	NR	NR
Olsen 2020[11]	RCT (1b)	51	NR (18-68)	M (18); F (33)	NR	2.12	Alopecia areata	NR	NR	NR	Ruxolitinib 1.5% [cream, BID] (51)	57.3/ 28.66	-50%	CR (8); PR (3); NOR (40)	180	NR	NR	NR
Putterman 2018[12]	CAS (4)	1	7	F	NR	3	Alopecia universalis	CS	NR	NR	Tofacitinib 2% [ointment, BID] (1)	100/ 83	-17%	PR (1)	NR	N (1)	NR	NR
Putterman 2018[12]	CAS (4)	1	15	F	NR	2	Alopecia totalis	CS	NR	NR	Tofacitinib 2% [ointment, BID] (1)	70/ 80	-14.3%	NOR (1)	NR	N (1)	NR	NR
Putterman 2018[12]	CAS (4)	1	16	F	NR	8	Alopecia universalis	CS	NR	NR	Tofacitinib 2% [ointment, BID] (1)	100/ 100	0%	NOR (1)	NR	N (1)	NR	NR
Putterman 2018[12]	CAS (4)	1	14	M	NR	9	Alopecia universalis	CS	NR	NR	Tofacitinib 2% [ointment, BID] (1)	100/ 10.5	-89.5%	PR (1)	NR	N (1)	NR	NR
Putterman 2018[12]	CAS (4)	1	12	F	NR	2.5	Alopecia areata	CS	NR	NR	Tofacitinib 2% [ointment, BID] (1)	15/ 10	-33.3%	PR (1)	NR	N (1)	NR	NR
Putterman 2018[12]	CAS (4)	1	6	F	NR	3	Alopecia universalis	CS	NR	NR	Tofacitinib 2% [ointment, BID] (1)	100/ 10	-90%	PR (1)	NR	N (1)	NR	NR
Putterman 2018[12]	CAS (4)	1	15	F	NR	4	Alopecia universalis	CS	NR	NR	Tofacitinib 2% [ointment, BID] (1)	87/ 61	-29.9%	PR (1)	NR	N (1)	NR	NR
Putterman 2018[12]	CAS (4)	1	11	F	NR	10	Alopecia totalis	CS	NR	NR	Tofacitinib 2% [ointment, BID] (1)	96/ 4	-95.8%	PR (1)	NR	N (1)	NR	NR
Putterman 2018[12]	CAS (4)	1	11	F	NR	6	Alopecia universalis	CS	NR	NR	Tofacitinib 2% [ointment, BID] (1)	77/ 53	-31.2%	PR (1)	NR	N (1)	NR	NR
Putterman 2018[12]	CAS (4)	1	15	F	NR	3	Alopecia totalis	CS	NR	NR	Tofacitinib 2% [ointment, BID] (1)	80/ 80	0%	NOR (1)	NR	N (1)	NR	NR
Putterman 2018[12]	CAS (4)	1	4	M	NR	3	Alopecia totalis	CS	NR	NR	Tofacitinib 2% [ointment, BID] (1)	100/ 78.5	-21.5%	PR (1)	NR	N (1)	NR	NR

References:

1. Bayart CB, DeNiro KL, Brichta L, Craiglow BG, Sidbury R. Topical Janus kinase inhibitors for the treatment of pediatric alopecia areata. *J Am Acad Dermatol*. 2017;77(1):167-170. doi:10.1016/j.jaad.2017.03.024

2. Bokhari L, Sinclair R. Treatment of alopecia universalis with topical Janus kinase inhibitors - a double blind, placebo, and active controlled pilot study. *Int J Dermatol*. 2018;57(12):1464-1470. doi:10.1111/ijd.14192

3. Cheng MW, Kehl A, Worswick S, Goh C. Successful treatment of severe alopecia areata with oral or topical tofacitinib. *J Drugs Dermatol*. 2018;17(7):800-803.

4. Craiglow BG, Tavares D, King BA. Topical ruxolitinib for the treatment of alopecia universalis. *JAMA Dermatol*. 2016;152(4):490-491. doi:10.1001/jamadermatol.2015.4445

5. Craiglow BG. Topical tofacitinib solution for the treatment of alopecia areata affecting eyelashes. *JAAD Case Rep*. 2018;4(10):988-989. doi:10.1016/j.jdcr.2018.07.018

6. Deeb M, Beach RA. A case of topical ruxolitinib treatment failure in alopecia areata. *J Cutan Med Surg*. 2017;21(6):562-563. doi:10.1177/1203475417716363

7. Ferreira SB, Ferreira RB, Scheinberg MA. Topical tofacitinib in treatment of alopecia areata. *Einstein (Sao Paulo)*. 2020;18:eAI5452. doi:10.31744/einstein_journal/2020ai5452

8. Kerkemeyer KLS, Sinclair RD, Bhoyrul B. Topical tofacitinib for the treatment of alopecia areata affecting facial hair. *Br J Dermatol*. 2021;185(3):677-679. doi:10.1111/bjd.20419

9. Liu LY, Craiglow BG, King BA. Tofacitinib 2% ointment, a topical Janus kinase inhibitor, for the treatment of alopecia areata: A pilot study of 10 patients. *J Am Acad Dermatol*. 2018;78(2):403-404.e1. doi:10.1016/j.jaad.2017.10.043

10. Mikhaylov D, Glickman JW, Del Duca E, et al. A phase 2a randomized vehicle-controlled multi-center study of the safety and efficacy of delgocitinib in subjects with moderate-to-severe alopecia areata. *Arch Derm Res*. Published online 2022. doi:10.1007/s00403-022-02336-0

11. Olsen EA, Kornacki D, Sun K, Hordinsky MK. Ruxolitinib cream for the treatment of patients with alopecia areata: A 2-part, double-blind, randomized, vehicle-controlled phase 2 study. *J Am Acad Dermatol*. 2020;82(2):412-419. doi:10.1016/j.jaad.2019.10.016

12. Putterman E, Castelo-Soccio L. Topical 2% tofacitinib for children with alopecia areata, alopecia totalis, and alopecia universalis. *J Am Acad Dermatol*. 2018;78(6):1207-1209.e1. doi:10.1016/j.jaad.2018.02.031
